# Laymen in the Department of Agriculture in England

**Published:** 1895-11

**Authors:** 


### LAYMEN IN THE DEPARTMENT OF AGRICULTURE IN ENGLAND.

English veterinarians are rightfully exercised over the practical wiping out of the Veterinary Department in connection with the Department of Agriculture, and the substitution of laymen for the places of experts and trained veterinarians. When all the world is reaching out to strengthen the veterinarian in his wide and important field of work, when every nation is tending strongly toward a proper recognition of the great service to be rendered by veterinary sanitary corps, and every large city in the New World agitating for a more thorough inspection of the meat- and milk-supply at every source and step of its production, it seems strange and unusual that a country as old and as far advanced in higher education as Great Britain should be stepping backward, and daring enough to imperil such a great source of wealth and sustenance to so large a number of her subjects, at a time when economy of resources and prevention of losses is the watchword of the hour the world over.

---

CHICAGO newspapers are strongly condemning the lax regulations which allow the sale of adulterated and impure milk among her people. Has Chicago's City Board of Health a veterinarian attached to its staff? Wake up, veterinarians, and tell your people what they have accomplished with such assistance in Philadelphia, Boston, New York, and are now gaining in Kansas City and many other places.

---

ALL honor to the Kansas City Veterinary College in her decisive step, that after January 1, 1896, she will be among the compulsory three-year colleges. This movement will inure to her benefit in many ways and demonstrates an honest and courageous example of the convictions of her founders and supporters. We heartily congratulate her on behalf of the veterinary profession of North America. Who will lead next in this grand movement?

---

THE Pennsylvania State Board of Veterinary Medical Examiners will meet at Harrisburg on November 14th, at 4 P.M., for the purpose of organization and election of officers.

